# Top-down, decoupled control of constitutive parameters in electromagnetic metamaterials with dielectric resonators of internal anisotropy

**DOI:** 10.1038/srep42447

**Published:** 2017-02-10

**Authors:** Sukmo Koo, Daniel R. Mason, Yunjung Kim, Namkyoo Park

**Affiliations:** 1 Photonic Systems Laboratory, Department of Electrical and Computer Engineering, Seoul National University, Seoul 08826, Korea

## Abstract

A meta-atom platform providing decoupled tuning for the constitutive wave parameters remains as a challenging problem, since the proposition of Pendry. Here we propose an electromagnetic meta-atom design of internal anisotropy (*ε*_*r*_ ≠ *ε*_*θ*_), as a pathway for decoupling of the effective- permittivity *ε*_eff_ and permeability *μ*_eff_. Deriving effective parameters for anisotropic meta-atom from the first principles, and then subsequent inverse-solving the obtained decoupled solution for a target set of *ε*_eff_ and *μ*_eff_, we also achieve an analytic, top-down determination for the internal structure of a meta-atom. To realize the anisotropy from isotropic materials, a particle of spatial permittivity modulation in *r* or *θ* direction is proposed. As an application example, a matched zero index dielectric meta-atom is demonstrated, to enable the super-funneling of a 50*λ*-wide flux through a sub-*λ* slit; unharnessing the flux collection limit dictated by the *λ*-zone.

Electromagnetic metamaterials exhibiting naturally non-occurring refractive indices^1^,^2^,^3^,^4^,^5^,^6^,^7^,^8^,^9^,^10^,^11^,^12^,^13^,^14^,^15^,^16^,^17^,^18^,^19^,^20^,^21^,^22^,^23^,^24^ and their application to exotic forms of wave manipulation^25^,^26^,^27^,^28^,^29^ have become one of the hottest research topics. Because electromagnetic metamaterials manifest their properties through electromagnetic couplings to the far-field, design strategies have been focused on the realization of designer electric (*ε*_eff_) and magnetic (*μ*_eff_) dipolar responses, with the engineering and assembly of metallic or dielectric building blocks. Nonetheless, in most cases, the realization of exotic *ε*_eff_ and *μ*_eff_ has been achieved via the simple combination of elementary resonators in a non-isotropic and polarization-dependent form^1^,^2^,^25^, at the same time based on retro-fit, bottom-up approaches - where the building blocks are initially proposed and the subsequent design is carried out through a series of iterations and guesswork.

Meanwhile, although the decoupling of those fundamental constitutive wave parameters has been envisaged as an ideal platform toward the top-down and deterministic design of the metamaterial (Pendry *et al*.^26^), its feasibility has not been treated until recently. With the presence of inherent cross-coupling terms between constitutive parameters (as shown for *ε*_*eff*_ and *μ*_eff_ in electromagnetics^3^, or 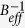 and *ρ*_eff_ in acoustics^30^), it is not always straightforward to achieve the decoupling of wave parameters. Elastic metamaterial for decoupling of density and stiffness in positive^31^ or negative^32^ parameter regime, sub skin-depth metallic particles of decoupled permittivity and permeability for positive high refractive index^4^,^5^, and lastly the acoustic omni meta-atom^30^ addressing the octant space of wave parameters have been demonstrated; yet existence of the meta-atom platforms which could access both positive and negative values of parameters, in the general spectrum of electromagnetic wave is not evident at this stage. Moreover, we note that the design of electromagnetic metamaterial has been largely based on metallic inclusions of well-defined current paths, meanwhile the intrinsic loss of metals makes dielectric metamaterial^1^,^6^,^7^,^8^,^9^,^10^ a more favorable option.

Here, we propose a new platform for electromagnetic metamaterial: a meta-atom of decoupled constitutive parameters, enabling top-down design - where the target *ε*_eff_ and *μ*_eff_ are first specified and the design parameters are subsequently determined, all the while using readily available lossless dielectrics. We first analytically solve the problem of decoupled effective- permittivity *ε*_eff_ and permeability *μ*_eff_, by introducing a hypothetical meta-atom of internal anisotropic susceptibility *χ*_*r*_ ≠ *χ*_*θ*_; for their axis are set in conform to the electric- and magnetic- characteristic movements of the electron, or equivalently to their corresponding dipole moments *p*_*r*_ (*χ*_*r*_) and *m*_*z*_ (*χ*_*θ*_) (Fig. 1). We then realize top-down design for the internal structure of a meta-atom, by inversely-solving the decoupled equation to get the required *χ*_*r*_ and *χ*_*θ*_ (or *ε*_*r*_ and *ε*_*θ*_) for targeted *ε*_eff_ and *μ*_eff_. Finally, a meta-atom implementation based on isotropic materials of spatial (*r, θ*) permittivity modulation is proposed to realize the required (*ε*_*r*_, *ε*_*θ*_) for a matched zero index, along with the demonstration of the super-funneling for a 50*λ*-wide flux through a sub-*λ* slit.

## Results

### Structure of the meta-atom for decoupled permittivity and permeability

We consider the two dimensional problem shown in Fig. 2(a); where a transverse electric (TE) plane wave is incident onto a cylindrical particle of radius *R* with split *ε*_*r*_, *ε*_*θ*_ anisotropy. To derive *ε*_eff_ and *μ*_eff_, we start from the zeroth order expressions for the electric and magnetic polarizabilities (*α*_*e*_ and *α*_*m*_)^11^ of the isolated particle,


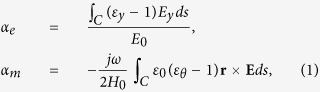


where *ε*_*y*_ and *ε*_*θ*_ being the permittivity of the particle along the *y* and *θ* direction respectively, and the integration is taken over the particle cross section *C*. It is noted that in Eq. (1) we treat only the dipole polarizability terms, with an implicit assumption of long-wavelength approximation. Solving the wave equation in polar coordinate for the general solution of **E** and **H** (details in Supplementary Information), and then keeping only the lowest order terms (within good approximation under *ε*_*r*_, *ε*_*θ*_ ≫ 1), we achieve analytical solutions for *α*_*e*_ and *α*_*m*_,


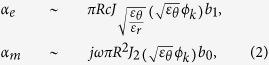


where *ϕ*_*k*_ = *k*_0_*R* and *b*_*n*_ being the coefficients of Bessel-Fourier expansions [Supplementary Eq. (S3)].

From *α*_*e*_ and *α*_*m*_ of the isolated particle (2), it is then straightforward to calculate *ε*_eff_ and *μ*_eff_ for a square lattice of meta-atoms. Using the mixing formula^12^ we arrive,


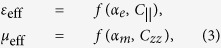


where 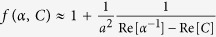 and 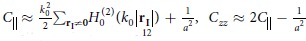 are the dynamic interaction constants with periodicity *a*, and a lattice point vector **r**_**I**_^12^.

**Figure 1:**
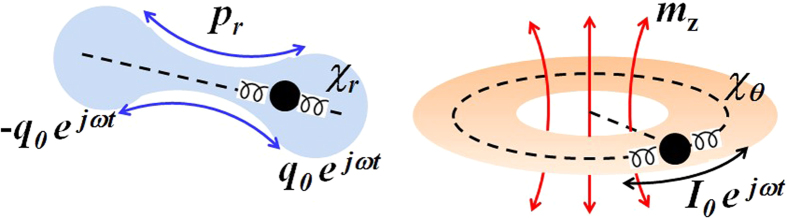
Physical origin of the electron induced electric (left) and magnetic (right) dipole moments of a classical atom.

**Figure 2:**
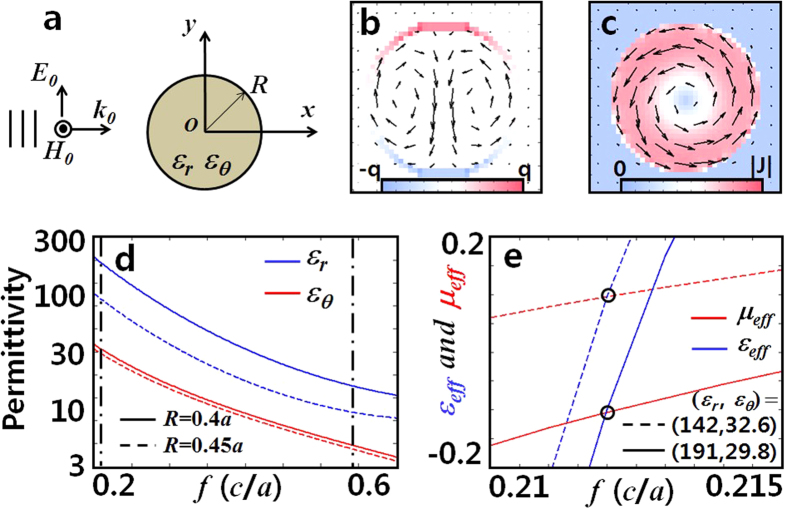
(**a**) Schematic of the anisotropic meta-atom illuminated by TE plane wave. (**b**) Charge and (**c**) current distribution at the electric and magnetic resonance frequencies, respectively (arrows denote current flow). (**d**) Calculated *ε*_*r*_ and *ε*_*θ*_ values that give matched zero index property (solid: *R* = 0.4*a*, dashed: *R* = 0.45*a*). (**e**) *ε*_eff_ and *μ*_eff_ tunability including demonstration of matched index property (*n*_eff_ = ±0.1).

Inspection of Eqs (2) and (3) clearly shows the dependence of *μ*_eff_ only on *ε*_*θ*_, or complete decoupling of *μ*_eff_ (or *α*_*m*_) from *ε*_*r*_, which supports separate tunability of *ε*_eff_ and *μ*_eff_ from the adjustment of *ε*_*r*_ and *ε*_*θ*_; confirming the proposed ansatzs based on anisotropic susceptibility in relation to the respective current patterns exhibited by the electric and magnetic modes [Figs 1 and 2(b,c)]. Because the obtained decoupling condition *α*_*e*_(*ε*_*r*_, *ε*_*θ*_) and *α*_*m*_(*ε*_*θ*_) are based on (2) - which are expanded from (1), the condition of decoupling becomes to follow the generic constraint of long-wavelength approximation in metamaterial applications; lattice period (*a*)/wavelength (*λ/n*_eff_) ~ 1/10. For example, with *a* = 5*λ*, the validity of our approximation would hold till |*n*_eff_| < 0.5. We also note, Eq. (3) works well in the low index regime (*n*_eff_ ≪ *λ*/2*a*^12^), while *ε*_eff_ and *μ*_eff_ of high values also always can be determined from S-matrix parameters^13^.

Focusing here on the low index case, we now proceed to *inverse*-solve the problem of (3), in order to determine required *ε*_*r*_ (*α*_*e*_) and *ε*_*θ*_ (*α*_*e*_, *α*_*m*_) by using (2), from *target ε*_eff_ and *μ*_eff_. Meanwhile the complete solution with Bessel-Fourier series (Supplementary Eq. (S3)) can also be used, here we show the simpler form of first-order approximated solution, near the first zeros of the Bessel functions^11^ (see Supplementary Information for details). For example, by specifying target *ε*_eff_ and *μ*_eff_ equal to 0, we arrive to a set of simple and intuitive relations which are used to calculate the required values of *ε*_*r*_ and *ε*_*θ*_ for the matched zero index;


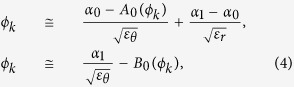


where *A*_0_(*ϕ*_*k*_) = *A(ε*_eff_ = 0; *ϕ*_*k*_) and *B*_0_(*ϕ*_*k*_) = *B(μ*_eff_ = 0; *ϕ*_*k*_) are slowly-varying functions of *ϕ*_*k*_ (Supplementary Eq. (S6) and Supplementary Fig. S1), and *α*_0_ (~2.405), *α*_1_ (~3.831) are the first zeros of the zeroth and first order Bessel functions. Again, for a given particle radius and frequency *ϕ*_*k*_ = *k*_0_*R*, the achievement of *μ*_eff_ = 0 from the *single* parameter *ε*_*θ*_ is evident from *B(μ*_eff_ = 0; *ϕ*_*k*_) in Eq. (4). Subsequent realization of *ε*_eff_ = 0 from the determination of *ε*_*r*_ is made then using *A(ε*_eff_ = 0; *ϕ*_*k*_) in (4). In Fig. 2(d) we show the solution obtained with Supplementary Eq. (S5), giving the values of (*ε*_*r*_, *ε*_*θ*_) that support a matched zero index at different target frequencies; for the particles of normalized radius *R* = 0.4*a* (solid lines) and 0.45*a* (dashed lines), of periodicity (*a*). The required (*ε*_*r*_, *ε*_*θ*_) value set depends on the frequency and particle size, and get smaller as either *f* or *R* increase. We also demonstrate in Fig. 2(e) the tunability of *ε*_eff_ and *μ*_eff_ by addressing matched index properties at *n*_eff_ = ±0.1, again, with the use of (3). It is worth to note that in all cases, the required *ε*_*r*_ is greater than *ε*_*θ*_, red-shifting the usually higher energy electric dipole resonance (*ε*_*r*_, *ε*_*θ*_) closer toward the lower energy magnetic dipole resonance (*ε*_*θ*_).

### Dielectric implementation of the anisotropic meta-atom

**Figure 3:**
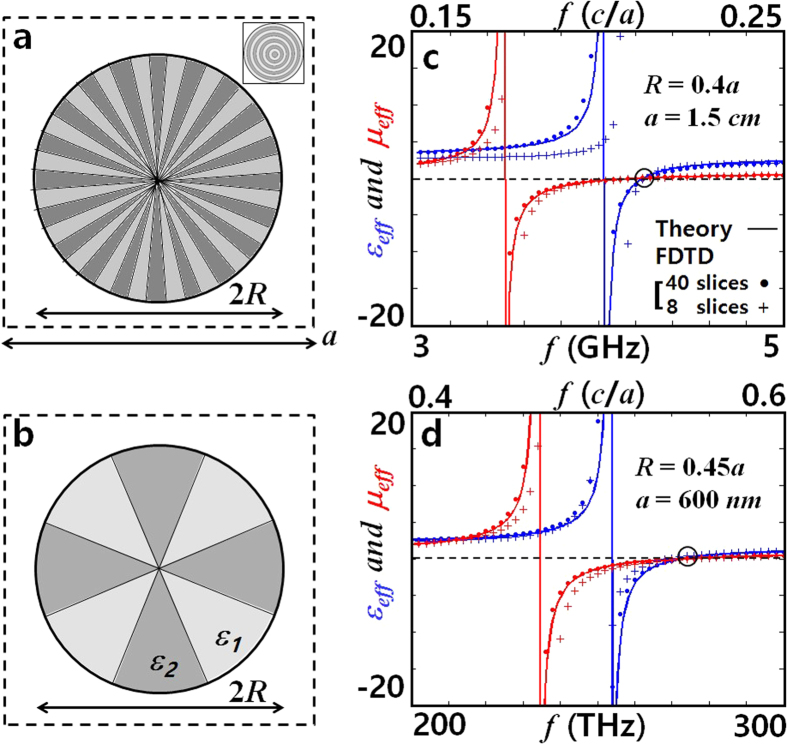
Anisotropic meta-atoms (*ε*_*r*_ ≠ *ε*_*θ*_) of nano-pizza cut geometry with (**a**) 40 slices and (**b**) 8 slices. (inset in (a) shows an example of nano-donut). Calculated *ε*_eff_ and *μ*_eff_ for meta-atoms lattice of radius (**c**) *R* = 0.4*a (a* = 1.5 cm), and (**d**) *R* = 0.45*a (a* = 600 nm). By appropriate choice of *a*, the operating frequency is determined. Circles indicate the frequencies of operation at matched zero index and horizontal dashed lines indicate *ε*_eff_ = 0 and *μ*_eff_ = 0. Excellent agreement between the theory and numerical results (for both 40 slice and 8 slice implementations), especially near the matched zero index, are observed.

To realize the set of required *ε*_*r*_ and *ε*_*θ*_ from isotropic materials, we spatially modulate the permittivity inside the particle along a given axis (**r** or **θ**). A proposed structure of nano-pizza cross-section is shown in Fig. 3(a). Extending the concept of average permittivity^33^ from Gauss’ law in polar coordinates we obtain,


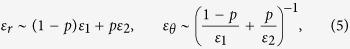


where *ε*_1_, *ε*_2_ (*ε*_1_ < *ε*_2_) are the permittivities of constituent dielectrics shown in Fig. 3(a), and *p* is the fill factor of slices containing *ε*_2_. While it is also possible to design the meta-atom for fixed *ε*_1_ (1, for example) by changing *p* and *ε*_2_, we here focus on the case of fill factor *p* = 0.5, without any loss of generality (design example with *p* = 0.83, for *ε*_1_ = 1 (air) and *ε*_2_ = 12.25 (silicon) is shown in the Supplementary Information). On the other hand, as the arithmetic mean is always larger than the harmonic mean in (5), the condition of *ε*_*r*_ ≥ *ε*_*θ*_ for matched zero index realization [Fig. 2(d)] is only met with nano-pizza cross-section geometry [we note, *ε*_*θ*_ ≥ *ε*_*r*_ for the nano-donut cross-section - inset of Fig. 3(a)]. Using (5) for the pair (*ε*_*r*_, *ε*_*θ*_) = (166, 31.1) [giving zero index at *f* = 0.212 *c/a* (4.24 GHz for *a* = 1.5 cm) and *R* = 0.4*a* from Fig. 2(d)], we obtain (*ε*_1_, *ε*_2_) = (16.32, 315.8).

In Fig. 3(c) we show plots of *ε*_eff_ (*α*_*e*_) and *μ*_eff_ (*α*_*m*_) obtained from Eq. (3), with *α*_*e*_ and *α*_*m*_ analytically obtained from Eq. (2) (solid lines), and also numerically obtained using Eq. (1) (solid dots) from the result of finite difference time domain simulations of a 40-slice structure. Near the zero-index, an almost perfect fit with less than 1% frequency error was obtained from that predicted by Supplementary Eq. (S5). In consideration of fabrication complexity, a structure with reduced number of slices has also been tested [Fig. 3(b)]. Even though the calculation of (*ε*_*r*_, *ε*_*θ*_) from (*p*; *ε*_1_, *ε*_2_) started to deviate from Eq. (5) when the size of slices was increased, it was still possible to determine (*ε*_1_, *ε*_2_) = (15.12, 171.9) providing a matched zero index for the 8-slice structure at *f* = 0.212 *c/a* [marked with ‘+’ symbol in Fig. 3(c)], by using few Newton iterations for the zero-index frequency deviation. It is noted that this value determined from the mixing formula (3) is in excellent agreement with exact values of (*ε*_1_, *ε*_2_) = (14.53, 179.2) extracted from S-matrix parameters^13^.

It is emphasized that, experimentally available, smaller permittivity values using Si and SiO_2_ for example [40 slices: (*ε*_1_, *ε*_2_) = (2.43, 15.13), 8 slices: (2.22, 12.96)], can be readily accessed by increasing the radius *R* of the particle to 0.45*a*, to give matched zero index at *f* = 0.546 *c/a* (e. g., *λ* = 1100 nm for *a* = 600 nm) [Fig. 3(d)]. The designs with 2D-slab structure (of height = 2*λ*, at GHz operation frequency) and a void at the particle center region are also discussed in Supplementary Information and Figures.

### Realization of the zero index super funneling through a subwavelength slit

**Figure 4:**
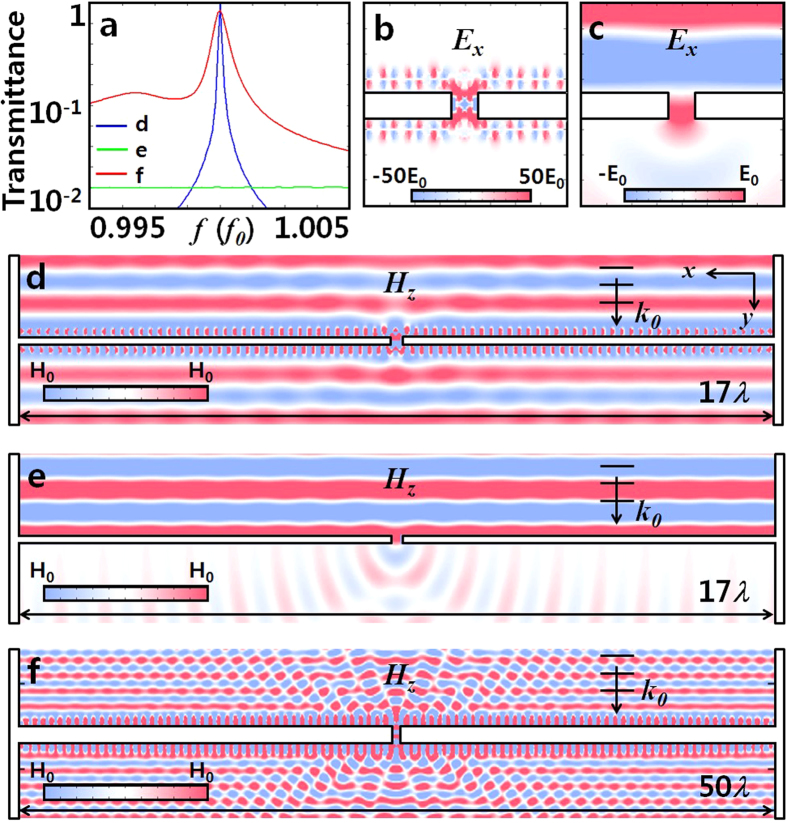
(**a**) Transmission spectra of the slit; without (green) and with matched zero-index meta-atom array of (blue) high index (*ε*_1_, *ε*_2_) = (14.53, 179.2) and (red) low index (2.22, 12.96) materials. *f*_0_ is the frequency of matched zero index. (**b**,**c**) *E*_*x*_ field pattern near the nanoslit at *f*_0_; (**b**) with and (**c**) without the zero-index meta-atom array. The near field pattern shows the associated dramatic enhancement of the electric field with the coating of matched zero index meta-atoms. (**d**–**f**) *H*_*z*_ field pattern of the slit at *f*_0_ (**d**) without, (**e**) with high-index, and (**f**) with low-index meta-atom array. Slit width; 0.21*λ* (**d**,**e**) and 0.55*λ* (**f**).

Using the matched zero index, we now investigate the problem of extraordinary optical transmission (EOT)^34^,^35^,^36^,^37^,^38^, for which the maximum field enhancement is limited by the *λ*-zone^34^. Applications of zero index tunneling have been demonstrated in the past^11^,^14^,^15^,^16^, yet the possibility of EOT beyond the *λ*-zone has not been investigated. A perfect electric conductor (PEC) having sub-wavelength (0.21*λ*) slit, of flux reception width far larger (17*λ*) than the *λ*-zone has been tested, with the application of single-layer matched zero index meta-atoms (*ε*_eff_ = *μ*_eff_ = 0, *f* = 0.212 *c/a*) covering the input/output regions of PEC. It is important to note that the tuning of meta-atom near the slit gap is necessary since the effective medium theory starts to deviates with the introduction of the metal slit in the meta-atom array, breaking the periodicity of the lattice. The detailed tuning procedure is described in the Supplementary Information. The transmittance of the matched zero-index meta-atom nanoslit shows almost perfect transmittance of 0.97 [Fig. 4(a,d)], a ~50 times increase compared to the slit without zero-index meta-atom coating [Fig. 4(a,e)]; demonstrating the super-funneling of flux which is 17 times greater than the *λ*-zone. A low-index 8-slice structure [(*ε*_1_, *ε*_2_) = (2.22, 12.96) at *R* = 0.45*a* providing matched zero index at *f* = 0.546 *c/a,* see Fig. 3(d)] over much larger flux reception area (50*λ*) also has been tested, to compensate for a factor of ~90 channel width variation (50*λ* to 0.55*λ*). A transmittance of 0.85 was achieved, showing the super-funneling of 42*λ*-flux (50*λ* · 0.85) through the meta-atom coated slit [Fig. 4(a,f)].

## Discussion

To summarize, a hypothetical meta-atom of internal (*r, θ*) anisotropy has been proposed. Introducing the split- symmetry of susceptibility *χ*_*r*_ ≠ *χ*_*θ*_ conforming to the orthogonal axes of current pathways of the respective electric- and magnetic- dipoles, we show analytically the decoupling and separate tunability of *ε*_eff_ and *μ*_eff._ The desired target optical response *ε*_eff_ and *μ*_eff_ are provided by top-down, analytically determined *ε*_*r*_ and *ε*_*θ*_ values, which are readily achieved with conventional isotropic materials in radial- or angular- anisotropic spatial arrangements. We note that, our approach widens the scope of metamaterial design; offering a top-down optical response (including both matched zero and negative index) from lossless dielectrics, meanwhile lifting the stringent restrictions of accidental degeneracy^6^ which itself was limited to matched zero index at fixed frequency. In an application to EOT, utilizing a single layer of matched zero index meta-atoms, we demonstrated for the first time a super-funneling of electromagnetic flux, overcoming the usual *λ*-zone limit by two orders. Our proposal of coordinate-conforming anisotropy for decoupling the electric and magnetic responses and thus the separate control of *ε*_eff_ and *μ*_eff_ should be applicable to elementary resonators in other exotic coordinate systems compliant to current pathways of chosen electric/magnetic resonances. We expect future development of other anisotropic meta-atom families based on our approach.

## Additional Information

**How to cite this article:** Koo, S. *et al*. Top-down, decoupled control of constitutive parameters in electromagnetic metamaterials with dielectric resonators of internal anisotropy. *Sci. Rep.* **7**, 42447; doi: 10.1038/srep42447 (2017).

**Publisher's note:** Springer Nature remains neutral with regard to jurisdictional claims in published maps and institutional affiliations.

## Supplementary Material

Supplementary Information
