# Supplementary material for: Top-down, decoupled control of constitutive parameters in electromagnetic metamaterials with dielectric resonators of internal anisotropy
Source: Sci Rep. 2017 Feb 10;7:42447. doi: 10.1038/srep42447 (PMC5301205; doi:10.1038/srep42447)
Supplement: Supplementary Information [file srep42447-s1.pdf]

Supplementary Information of "Top-down, decoupled control of constitutive parameters in electromagnetic metamaterials with dielectric resonators of internal anisotropy"

Sukmo Koo[1,2], Daniel R. Mason[1], Yunjung Kim[1] and Namkyoo Park[1]*

[1]*Photonic Systems Laboratory, Department of Electrical and Computer Engineering, Seoul National University, Seoul, Korea 08826*

[2]*Current address: Moore Laboratory, Department of Medical Engineering, California Institute of Technology, Pasadena, California, USA 91125*

**E-mail address for correspondence: nkpark@snu.ac.kr*

Solution of the wave equations for an isolated anisotropic meta-atom

To treat the (r, θ) anisotropic meta-atom shown in manuscript Fig. 2(a), we use the auxiliary vector potential **F** as **D** = –∇ × **F** [1], where **D** is the electric displacement vector. The TE mode scalar wave equation for F_z can then be written as [1],

$$r\frac{\partial}{\partial r}\left(r\frac{\partial F_z}{\partial r}\right)+\frac{\varepsilon_r}{\varepsilon_\theta}\frac{\partial^2 F_z}{\partial\theta^2}+\omega^2\mu_0\varepsilon_0\varepsilon_\theta r^2 F_z=0. \tag{S1}$$

Solving Eq. (S1) by separation of variables, the general solution for F_z is expanded in terms of the Bessel-Fourier series,

$$F_z=\begin{cases} H_0\sum_{n=0}^{\infty} b_n J_{\sqrt{\frac{\varepsilon_\theta}{\varepsilon_r}}n}(\sqrt{\varepsilon_\theta}k_0 r)\cos(n\theta) & (r<R) \\ H_0\sum_{n=0}^{\infty} a_n H_n^{(2)}(k_0 r)\cos(n\theta)+F_{z0} & (r>R)\end{cases}, \tag{S2}$$

where $F_{z0}=-\frac{H_0}{j\omega}\sum_{n=0}^{\infty}(-j)^n(2-\delta_{n0})J_n(k_0 r)\cos(n\theta)$, δ_{ij} is Kronecker delta, H_0 is the incident magnetic field amplitude, $k_\theta=(\varepsilon_\theta)^{1/2}k_0$, J_n and $H_n^{(2)}$ are the Bessel and Hankel (second kind) functions. From **D** = –∇ × **F** and $-j\omega\mu_0$**H** = ∇ × **E**, the distribution of **E** and **H** can be calculated.

The polarizabilities α_e and α_m in Eq. (1) then can be expressed in terms of Bessel-Fourier series, while the coefficients b_n are determined from the boundary conditions for tangential **E** and **H** at $r = R$ (where $\phi_k = k_0R$);

$$b_n = \frac{2(2-\delta_{n0})(-j)^n / \pi\omega\phi_k}{J_{\sqrt{\frac{\varepsilon_\theta}{\varepsilon_r}}n}(\sqrt{\varepsilon_\theta}\phi_k)H'^{(2)}_n(\phi_k) - \frac{1}{\sqrt{\varepsilon_\theta}}J'_{\sqrt{\frac{\varepsilon_\theta}{\varepsilon_r}}n}(\sqrt{\varepsilon_\theta}\phi_k)H^{(2)}_n(\phi_k)}. \tag{S3}$$

Inverse-solving of Eq. (3) for the top-down determination of (ε_r, ε_θ) from target (ε_{eff}, μ_{eff})

To solve the inverse problem of Eq. (3) in the manuscript, we first simplify α_e and α_m in Eq. (2). Expressing α_e and α_m in terms of $J_{\sqrt{\varepsilon_\theta/\varepsilon_r}}(\sqrt{\varepsilon_\theta}\phi_k)$ and $J_1(\sqrt{\varepsilon_\theta}\phi_k)$ by using recurrence relations of Bessel functions $J_{l+1}(x) = (l/x)J_l(x) - J'_l(x)$ and $J_{l-1}(x) = [(l+1)/x]J_l(x) + J'_l(x)$ we arrive to,

$$\alpha_e = -\frac{j4Rc}{\omega\phi_k}\frac{1}{H'^{(2)}_1(\phi_k) - H^{(2)}_1(\phi_k)[J'_{\sqrt{\frac{\varepsilon_\theta}{\varepsilon_r}}}(\sqrt{\varepsilon_\theta}\phi_k)]/[\sqrt{\varepsilon_\theta}J_{\sqrt{\frac{\varepsilon_\theta}{\varepsilon_r}}}(\sqrt{\varepsilon_\theta}\phi_k)]},$$
$$\alpha_m = \frac{j2R^2}{\phi_k}\frac{\frac{1}{\phi_k} - [\sqrt{\varepsilon_\theta}J'_1(\sqrt{\varepsilon_\theta}\phi_k)/J_1(\sqrt{\varepsilon_\theta}\phi_k)]}{[\frac{2}{\phi_k} + [\sqrt{\varepsilon_\theta}J'_1(\sqrt{\varepsilon_\theta}\phi_k)/J_1(\sqrt{\varepsilon_\theta}\phi_k)]]H'^{(2)}_0(\phi_k) + H^{(2)}_0(\phi_k)}. \tag{S4}$$

Using (S4), we now group all terms in ε_r and ε_θ on the left hand side of manuscript Eq. (3), to get,

$$[J_{\sqrt{\frac{\varepsilon_\theta}{\varepsilon_r}}}'(\sqrt{\varepsilon_\theta}\phi_k)]/[\sqrt{\varepsilon_\theta}J_{\sqrt{\frac{\varepsilon_\theta}{\varepsilon_r}}}(\sqrt{\varepsilon_\theta}\phi_k)] = A(\phi_k)^{-1}$$
$$[\sqrt{\varepsilon_\theta}J_1'(\sqrt{\varepsilon_\theta}\phi_k)]/[J_1(\sqrt{\varepsilon_\theta}\phi_k)] = B(\phi_k)^{-1} \tag{S5}$$

where $A(\phi_k)$ and $B(\phi_k)$ are slowly varying functions of ϕ_k shown in Supplementary Fig. S1.

$$A(\phi_k)^{-1} = \frac{4jRc}{\omega\phi_k H^{(2)}_1(\phi_k)}[\frac{1}{a^2(\varepsilon_{\text{eff}}-1)} + C_{\parallel}]^{-1} + \frac{H'^{(2)}_1(\phi_k)}{H^{(2)}_1(\phi_k)},$$
$$B(\phi_k)^{-1} = \frac{1}{\phi_k}[1 - \frac{\phi_k H^{(2)}_0(\phi_k) + 3H'^{(2)}_0(\phi_k)}{H'^{(2)}_0(\phi_k) + j2R^2[\frac{1}{a^2(\mu_{\text{eff}}-1)} + C_{zz}]}]. \tag{S6}$$

Taking the solution for $\phi_k = k_0R$ to first order $J_n(x) \sim J'_n(x)(x-\alpha_n)$ near the first zeros of the Bessel functions α_n [2], Eq. (S5) can be put in a simpler and intuitive form.

$$\phi_k \sim \frac{\alpha_0 - A(\phi_k)}{\sqrt{\varepsilon_\theta}} + \frac{\alpha_1 - \alpha_0}{\sqrt{\varepsilon_r}},$$
$$\phi_k \sim \frac{\alpha_1}{\sqrt{\varepsilon_\theta}} - B(\phi_k). \tag{S7}$$

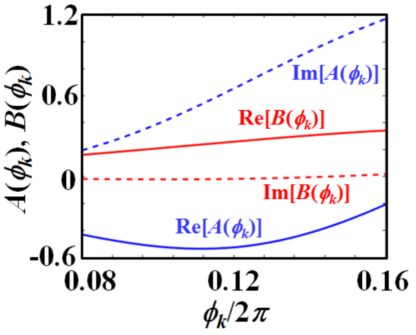

Supplementary Figure S1. Plot of $A(\phi_k)$ and $B(\phi_k)$ for $\varepsilon_{\mathrm{eff}} = \mu_{\mathrm{eff}} = 0$, plotted for $\phi_k = 0.08\times2\pi$ to $0.16\times2\pi$ (or f = 0.2 to 0.4 c/a. $R = 0.4a$)

Fine tuning of the zero index meta-atom near the subwavelength slit

It is important to note that the introduction of a metal slit in the array of matched zero-index meta-atom results in a deviation $\Delta\varepsilon$ and $\Delta\mu$ from zero, hindering the ideal unity transmission through the slit structure. To recover the matched zero index, the capability of independent fine control of $\varepsilon_{\mathrm{eff}}$ and μ_{eff}, as provided by our meta-atom is critical. For this, we focus on particles A and B (B') located at positions of abrupt change in the lateral PEC confinement [Supplementary. Fig. S2(a)].

Supplementary Fig. S2(b) shows slit-perturbed deviation $\Delta\varepsilon_{\mathrm{III}}$ from ideal zero, for the particle *array* at the output face [part III of the slit, Supplementary Fig. S2(a)]; before (dash-dot) and after (solid) the ε-tuning (ε_{2B} from 179.2 to 207.4) of the particle B (and B'). With the fine tuning of ε_{2B}, the nonzero Im($\Delta\varepsilon_{\mathrm{III}}$) at f = 0.212 c/a (marked with red circle symbol) has been successfully brought back to zero (marked with red triangle symbol). The deviation $\Delta\mu$ is negligible, with the absence of magnetic charge for PEC ($\mu = 1$, $\varepsilon = \infty$) leading to negligible distortion of the magnetic field [3,4]. As can be seen in Supplementary Fig. S2(d),(e), the effect of particle B (and B') tuning is clear in the removal of higher modal components. Subsequently, the remaining nonzero deviation of Re($\Delta\varepsilon_{\mathrm{III}}$) [Supplementary Fig. 2(b)] for particle arrays I and III are compensated with the Re($\Delta\varepsilon_{\mathrm{II}}$), from the fine-

tuning of particle A in region II; to achieve $\mathrm{Re}(\Delta\varepsilon_{\mathrm{II}}) + 2\cdot\mathrm{Re}(\Delta\varepsilon_{\mathrm{III}}) = 0$ (ε_{2A} from 179.2 to 168.0, from blue circle to triangle symbols, Supplementary Fig. S2(c)). It is worth to noting that transmission with larger number of in-/out-put layers worked slightly better in our test, but it was anyway necessary to fine-tune the meta-atom near the gap. To compensate for a factor of ~90 channel width variation (50λ to 0.55λ) with a low-index 8-slice structure, an increased number of tuning particles [A to G – see Supplementary Fig. S2(a)] were used to suppress higher order modal components of nonzero k_x.

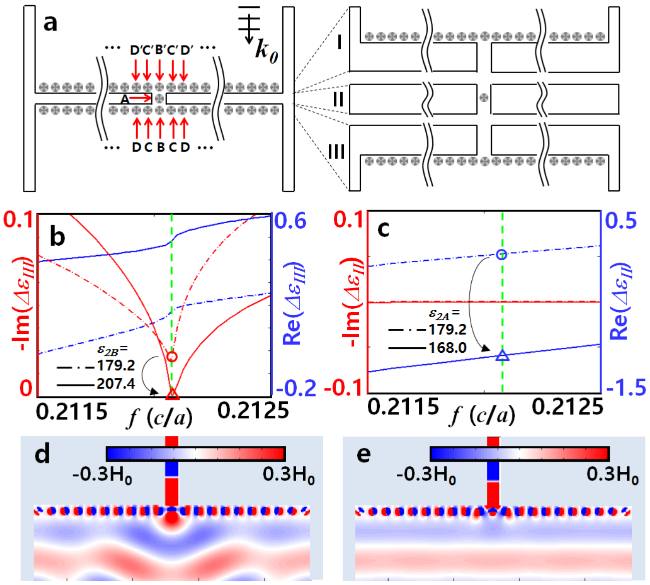

Supplementary Figure S2. (a) Schematic of the meta-atom coated slit structure (left), analyzed as an assembly of three parts (right). Deviation of effective permittivity $\Delta\varepsilon$ before (dash-dot) and after (solid) tuning of; (b) particle B in part III, and (c) particle A in part II. Extracted from the S-matrix parameters. Meta-atom of 8 slice structure (ε_1, ε_2) = (14.53, 179.2) was used. Green dash line denotes f = 0.212 c/a. (d), (e) Magnetic field pattern in the part III, (d) without, and (e) with the tuning of particle B near the gap.

Implementations in the form of 2D-slab design and void at the center region

The design used in the theoretical analysis (infinite cylinder, with fabrication unfriendly geometry at the center) need to be modified for the experimental implementation. First, following the generic approach - converting 2D photonic crystal design to the 2D-slab - we use a meta-atom slab of sufficient height (> 2λ, top and bottom covered with metal [5]), to secure negligible field variation along the z direction (see Supplementary Fig. S3(a)).

For λ = 4.1cm and a = 2 cm with the slab height of 8cm, Supplementary Fig. S3(b) shows the result of 3D FEM obtained effective- permittivity / permeability of the meta-atom slab. Assuming reasonable values of refractive index and fill factor (n_1 = 1.5, n_2 = 3.5, p = 2/3), matched zero index response is successfully realized at 7.3 GHz. It is worth to noting that the obtained ε_{eff} and μ_{eff} from 2D-slab (sloid lines) exhibit excellent overlap to those obtained for the infinite cylinder (ideal 2D design, dashed lines).

As well, to test the fabrication tolerance, we compared a structure with a void at the center (Supplementary Fig. S3(c)), against the ideal design without a void. Assuming Silicon (n = 3.5) and air as high- and low- index material, with p = 5/6 and a = 160 μm, the matched zero index response at 0.97 THz from the void structure (solid lines, Supplementary Fig. S3(d)) shows excellent match to the ideal 2D design without a void (dashed lines) for the μ_{eff}. It is worth to noting that, the minor shift in ε_{eff} could be readily compensated by simply applying observed offset to the target permittivity value in the design process.

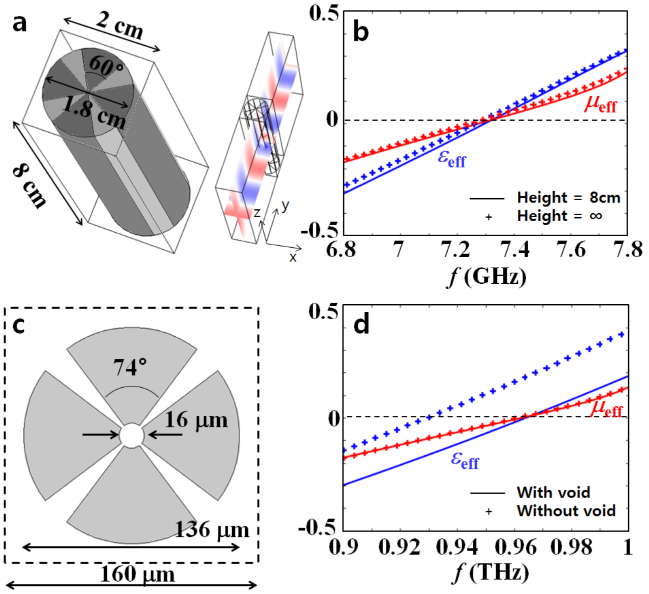

Supplementary Figure S3. (a) Schematic of the 2D-slab meta-atom. Inset shows field profiles (E_x) near the zero index frequency. (b) 3D-FEM calculated ε_{eff} and μ_{eff} in GHz regime (solid lines : 2D-slab, cross symbols : ideal 2D). (c) 2D meta-atom design with a void center. (d) FEM obtained ε_{eff} and μ_{eff} (solid lines : void structure, cross symbols : ideal 2D, without a void).

Supplementary References

1. Sheen, J. Time harmonic electromagnetic fields in an biaxial anisotropic medium. *J. of Electromagn. Waves and Appl.* **19**, 753-767 (2005).

2. Silveirinha, M. & Engheta, N. Design of matched zero-index metamaterials using nonmagnetic inclusions in epsilon-near-zero media. *Phys. Rev. B* **75**, 075119 (2007).

3. Seo, M. A. *et al.* Terahertz field enhancement by a metallic nano slit operating beyond the skin-depth limit. *Nat. Photon.* **3**, 152-156 (2009).

4. Koo, S., Kumar, M. S., Shin, J., Kim D. & Park, N. Extraordinary magnetic field enhancement with metallic nanowire: role of surface impedance in Babinet's principle for sub-skin-depth regime. *Phys. Rev. Lett.* **103**, 263901 (2009).

5. Huang, X., Lia, Y., Hang, Z. H., Zheng H. & Chan, C. T. Dirac cones induced by accidental degeneracy in photonic crystals and zero-refractive-index materials. *Nat. Materials* **10**, 582-586 (2011).
